# Identification of *Dietzia* Species in a Patient with Endophthalmitis following Penetrating Injury with Retained Intraocular Metallic Foreign Body

**DOI:** 10.1155/2018/3027846

**Published:** 2018-09-25

**Authors:** Jesintha Navaratnam, Lumnije Dedi, Andreas Myklebust Tjølsen, Ragnheiður Bragadóttir

**Affiliations:** ^1^Department of Ophthalmology, Oslo University Hospital, Oslo, Norway; ^2^University of Oslo, Oslo, Norway; ^3^Department of Microbiology, Oslo University Hospital, Oslo, Norway; ^4^Department of Radiology, Vestfold Hospital Trust, Tønsberg, Norway

## Abstract

To the best of our knowledge, we report the first case of *Dietzia* species in a patient with endophthalmitis. A 47-year-old man presented to the ophthalmology department with decreased vision, redness, and minimal pain in his right eye after a foreign body struck his eye following working using a hammer and chisel. Broad-spectrum polymerase chain reaction (PCR) and deoxyribonucleic acid (DNA) sequencing targeting 16S ribosomal ribonucleic acid-(rRNA-) encoding gene on an undiluted vitreous sample revealed 100% identity with GenBank sequences of *Dietzia* species including *D. natronolimnaea*, *D. dagingensis*, and *D. cercidiphylli*. The culture of the vitreous samples demonstrated the growth of Gram-positive cocci and polymorphic rods. The isolate from the culture was identified as *D. natronolimnaea* using matrix-assisted laser desorption/ionisation time-of-flight mass spectrometry (MALDI-TOF MS). The combination of surgical and medical treatment (pars plana vitrectomy and systemic and topical antibiotics) eradicated the infection successfully.

## 1. Introduction

Infectious endophthalmitis is a sight-threatening infection. The incidence of infectious endophthalmitis varies between 0.0075% and 1.05% [1–9]. It can be further categorized into exogenous and endogenous types. If a distant source of infection spread hematogenously, it gives rise to endogenous endophthalmitis. The exogenous endophthalmitis represents the most common type of endophthalmitis and results from direct inoculation of an organism as a complication following ocular surgery, penetrating ocular trauma with or without retained foreign body, intravitreal injections of medications, or extension of corneal infection. Coagulase-negative staphylococci represent the most common pathogen in endophthalmitis following cataract surgery and intravitreal injections, and *Bacillus cereus* causes the most cases of endophthalmitis following trauma [10]. *D. natronolimnaea* is an aerobically growing Gram- and catalase-positive actinomycete. A case report involving *D. natronolimnaea* in human disease describes a case of culture negative device-associated endocarditis [11]. However, in this case, *D. natronolimnaea* could not be differentiated from *D. cercidiphylli* strain by DNA sequencing of the excised atrial tissue.

## 2. Case Presentation

**Figure 1:**
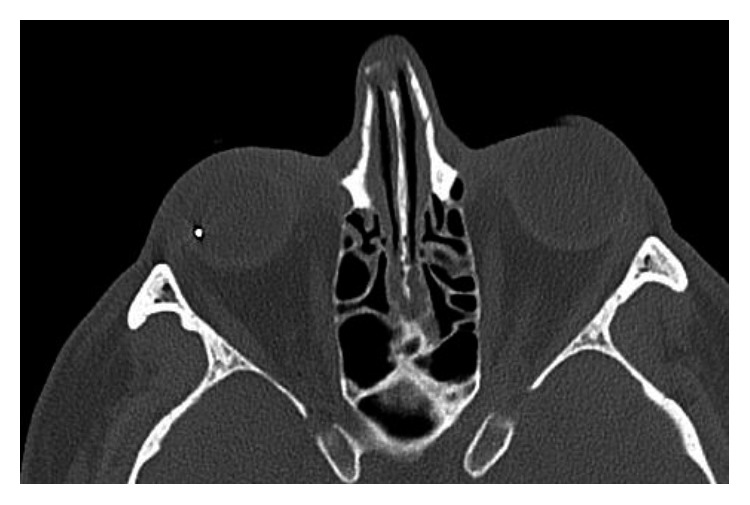
The computed tomography scan of the orbit demonstrates a metallic intraocular foreign body in the patient's right eye.

A 47-year-old Norwegian male presented at the general causality clinic with right eye irritation. Previously, the same day he had been working using a hammer and chisel to repair his car without any eye protection, and he thought a foreign body had struck his right eye. The examination of his right eye revealed congestion and laceration of the conjunctiva, and the general practitioner started treatment with a broad-spectrum topical antibiotic (chloramphenicol). Seven days following his first presentation to the general causality clinic, he woke up with decreased vision, redness, and minimal pain in his right eye, and he presented to the nearby ophthalmology department on the same day. The best-corrected visual acuity (BCVA) decimal had decreased from 1.0 to 0.7. The orbit computed tomography scans detected a metallic intraocular foreign body (Figure 1), and he was referred urgently to the Department of Ophthalmology at Oslo University Hospital for surgical removal of the foreign body. On arrival, the BCVA decimal had decreased from 0.7 to hand motion. The biomicroscopic examination revealed intense conjunctival and ciliary injection, most likely self-sealed conjunctival laceration, corneal oedema, 3+ anterior chamber cells with fibrin, and a thin layer of hypopyon in the anterior chamber and posterior synechiae. A layer of fibrin mesh covered the anterior surface of the lens. The changes in ocular media obscured the fundus view. The B-scan ultrasonography revealed an echogenic foreign body in the posterior vitreous cavity with dense vitreous opacities and attached retina and posterior vitreous. His left eye was unremarkable, and he was otherwise in good health. A clinical diagnosis of exogenous endophthalmitis secondary to penetrating eye injury with retained intraocular metallic foreign body was made.

He underwent an emergency 23-gauge pars plana vitrectomy with both undiluted and diluted vitreous biopsy and anterior chamber tap. The attempt to remove the intraocular foreign body was unsuccessful even after the removal of fibrin mesh layer covering the anterior surface of the lens due to poor surgical visualization of the posterior segment. At the end of the surgery, vancomycin (1 mg/0.1 ml), ceftazidime (2 mg/0.1 ml), and fungizone (0.00549 mg/0.1 ml) were administered intravitreally and gentamicin (20 mg/0.5 ml) subconjunctivally. He received treatment with topical steroids and antibiotics (Maxitrol eye drops consisting of dexamethasone, neomycin, and polymyxin B eye drops) hourly during daytime and intravenous cefuroxime (750 mg 3 times a day) postoperatively. In addition, he received oral steroids (prednisolone 60 mg daily) only for 2 days prior to repeated pars plana vitrectomy. Due to poor visualization of the posterior segment initially, he underwent surgical removal of the metallic intraocular foreign body (2 × 1.5 × 1 millimetres) 7 days after the first operation. The day after surgical removal of the foreign body, the intravenous cefuroxime was discontinued, and he was prescribed a 10-day course of ciprofloxacin (750 mg three times a day) peroral treatment. He continued with topical steroids and antibiotics (dexamethasone, neomycin, and polymyxin B eye drops) three times a day. The routine postoperative eye examination on the 9^th^ day following removal of the foreign body revealed asymptomatic rhegmatogenous retinal detachment from 10 to 2 o'clock with fovea on and with a BCVA decimal of 0.4. He underwent his third pars plana vitrectomy with gas tamponade. Three weeks following the retinal detachment repair, he reached a BCVA decimal of 0.8, and the retina was attached. Six months following the retinal detachment repair, he reached a BCVA decimal of 1.0.

### 2.1. Microbiological Analysis

Direct microscopy of the undiluted vitreous sample showed pleomorphic rods with coccoid or club-shaped appearance suggesting coryneform bacteria.

A broad PCR and DNA sequencing targeting 16S rRNA-encoding gene was performed on the undiluted vitreous body sample using EZI DNA tissue kit (Qiagen, Thermo Fisher Scientific, Hilden, Germany). The 5' half of the 16S rRNA gene was amplified by PCR. The PCR product was sequenced using BigDye Terminator Cycle Sequencing Kit (Thermo Fisher Scientific, Hilden, Germany). The bacterium was identified by searching the GenBank (the NIH genetic sequence database) with the obtained DNA sequence. The obtained 740-base pair DNA sequence revealed 100% identity with GenBank sequences of the *Dietzia* species including *D. natronolimnaea*, *D. dagingensis*, and *D. cercidiphylli*. Although, additional DNA sequencing on the rest of the 16S rRNA gene (total 1462 base pairs) was performed, and this did not give an unambiguous identification.

The undiluted and diluted vitreous samples were cultured on sheep blood agar and chocolate agar plates (Oslo University Hospital, Oslo, Norway). The plates were incubated at 35°C in 5% carbon dioxide (CO_2_) for 7 days. The growth of bacteria was detected after two days under aerobic incubation at 35°C in 5% CO_2_. Anaerobic culture using horse blood agar (Oslo University Hospital, Oslo, Norway) did not detect any bacterial growth, and yeast culture on Sabouraud agar (Oslo University Hospital, Oslo, Norway) did not reveal any growth. No bacterial or yeast growth was detected from the anterior chamber tap aspirate.

The Gram stain of the bacterial colonies demonstrated Gram-positive cocci and polymorphic rods. The isolate from the culture was identified as *D. natronolimnaea* using MALDI-TOF MS (MALDI Biotyper, Bruker Daltonics GmbH, Bremen, Germany). MALDI-TOF was unable to distinguish *D. natronolimnaea* from *D. dagingensis* and *D. cercidiphylli* because the MALDI-TOF database does not contain the sequences for the latter two species.

Antimicrobial susceptibility tests were performed on Mueller-Hinton agar (Oslo University Hospital, Oslo, Norway) using MIC test strips (Liofilchem, Teramo, Italy). There are no determined breakpoints for *Dietzia species*, and therefore, the results were reported with a minimum inhibitory concentration (MIC) value. The MIC values of ciprofloxacin, gentamycin, tetracycline, and vancomycin against *Dietzia* species were low, indicating that these antibiotics have clinical effect.

## 3. Discussion

Rainey et al. proposed the new classification of genus *Dietzia* in 1995, and they suggested that *Rhodococcus maris* should be reclassified into a new genus, *Dietzia* [12]. The main natural reservoirs for *Dietzia* species include soil and marine sediments. *D. natronolimnaea* was first isolated from an East African soda lake in Kenya by Duckworth et al. [13]. The importance of recently established genus *Dietzia* in medical conditions in human is slowly emerging. *D. maris* has been reported as an etiological agent in a total number of three patients. They presented with prosthetic hip infection [14], septicaemia [15], and aortic dissection secondary to aortitis [16]. This strain has also been detected from the skin of healthy individuals [17]. The strain *D. cinnamea* has been isolated from a perianal swab in a patient with bone marrow transplantation [18] and from a dog bite wound in an adult patient [19]. The authors in both case reports questioned the potential of *D. cinnamea* to cause infection. Jones et al. isolated *D. papillomatosis* from an immunocompromised patient with confluent and reticulated papillomatosis, which is a benign skin disorder [20]. Rammer et al. identified *D. papillomatosis* in blood culture in a 2-year-old child who presented with fever following revision of ventriculoperitoneal shunt inserted as treatment for syringomyelia [21]. Furthermore, *Dietzia* species has been isolated repeatedly in a hematological unit in the United Kingdom [22]. At present, the only report of *D. aurantiaca* from human clinical specimens is from a 24-year-old Swedish woman's cerebrospinal fluid [23]. In another case, an 87-year-old man developed cellulitis-like inflammation 10 months following permanent pacemaker insertion, and *Dietzia* species were detected [24]. The authors in the culture negative device-associated endocarditis involving *D. natronolimnaea* stated that the DNA sequence from the excised atrial tissue of the patient revealed *D. natronolimnaea* that shared 100% identity with *D. cercidiphylli* strain. Very few case reports describe identification of *Dietzia* species in human conditions. However, their role in causing infection should be elucidated in the future.

The identification of the infecting organism in exogenous endophthalmitis may be challenging. In a study from which bacteriologic data were obtained from postoperative endophthalmitis cases, only 291 out of 420 patients (69.3%) demonstrated microbial growth. [25] In this study, Gram-positive bacteria were isolated in 274 patients (94.2%).

The similarities in the Gram morphology and colony appearance displayed by *Dietzia* species and the more frequently encountered *Rhodococcus* species may lead to misinterpretation of *Dietzia* species as *Rhodococcus* species. This may implicate the underdiagnosis of *Dietzia* species-related infections in humans. Additionally, *D. natronolimnaea* may easily be missed, if cultured less than 72 hours and at incubation temperature at or above 37°C. The growth occurs at pH 6–10 with optimal growth at pH 9 and at salt concentrations up to 10% [22]. Previously, the diagnosis of *Dietzia* species may have been challenging due to the lack of accurate diagnostic tests. Although the DNA sequencing of 16S rRNA encoding gene assists in precise determination of species, some of the *Dietzia* species share 100% gene identity. In the patient described in this case report, the DNA sequencing of 16S rRNA encoding gene revealed 100% identity with GenBank sequences of *D. natronolimnaea*, *D. dagingensis*, and *D. cercidiphylli*. Similarly, Sudhindra et al. could not distinguish between the former two *Dietzia* species. The unique characteristic of mycolic acids in *Dietzia* may indicate a novel fatty biosynthesis [26]. This may aid in development of new accurate diagnostic laboratory tests. In future, increased awareness and development of accurate tests may assist in the diagnosis of *Dietzia* species infections.
